# Activation of ATP-sensitive potassium channels enhances DMT1-mediated iron uptake in SK-N-SH cells *in vitro*

**DOI:** 10.1038/srep33674

**Published:** 2016-09-20

**Authors:** Xixun Du, Huamin Xu, Limin Shi, Zhifeng Jiang, Ning Song, Hong Jiang, Junxia Xie

**Affiliations:** 1 Collaborative Innovation Center for Brain Science, Department of Physiology, Shandong Provincial Collaborative Innovation Center for Neurodegenerative Disorders, Key Laboratory of Pathogenesis and Prevention of Neurological Disorders and State Key Disciplines: Physiology, Medical College of Qingdao University, Qingdao, 266071, China

## Abstract

Iron importer divalent metal transporter 1 (DMT1) plays a crucial role in the nigal iron accumulation in Parkinson’s disease (PD). Membrane hyperpolarization is one of the factors that could affect its iron transport function. Besides iron, selective activation of the ATP-sensitive potassium (K_ATP_) channels also contributes to the vulnerability of dopaminergic neurons in PD. Interestingly, activation of K_ATP_ channels could induce membrane hyperpolarization. Therefore, it is of vital importance to study the effects of activation of K_ATP_ channels on DMT1-mediated iron uptake function. In the present study, activation of K_ATP_ channels by diazoxide resulted in the hyperpolarization of the membrane potential and increased DMT1-mediated iron uptake in SK-N-SH cells. This led to an increase in intracellular iron levels and a subsequent decrease in the mitochondrial membrane potential and an increase in ROS production. Delayed inactivation of the Fe^2+^-evoked currents by diazoxide was recorded by patch clamp in HEK293 cells, which demonstrated that diazoxide could prolonged DMT1-facilitated iron transport. While inhibition of K_ATP_ channels by glibenclamide could block ferrous iron influx and the subsequent cell damage. Overexpression of Kir6.2/SUR1 resulted in an increase in iron influx and intracellular iron levels, which was markedly increased after diazoxide treatment.

Divalent metal transporter 1 (DMT1) is a ferrous iron importer and plays an important role in both iron uptake and iron translocation from the endosome^1^. Increased iron contents are well documented in the substantia nigra (SN) of Parkinson’s disease (PD)^2^,^3^,^4^,^5^,^6^,^7^,^8^,^9^,^10^,^11^. Due to its toxic effect to produce highly reactive hydroxyl radicals by Fenton reaction, the accumulation of iron in the SN plays an important role in the degeneration of dopaminergic neurons. Increased expression of DMT1 might account for this selective nigral iron accumulation, which was found both in PD patients and animal models by our previous works, together with others^12^,^13^.

The iron transport function of DMT1 is not only based on its expression levels, but also dependent on its ability to transport, which could be enhanced by relatively lower pH and membrane potential hyperpolarization^12^,^14^. Besides iron insult to nigral dopaminergic neurons in PD, selective activation of the ATP-sensitive potassium (K_ATP_) channels also contributes to the differential vulnerability of dopaminergic neurons^15^,^16^. Interestingly, activation of these channels could induce membrane hyperpolarization due to ATP depletion and increased oxidative stress (ROS) in dopaminergic neurons. Since there is a high density of K_ATP_ channels in the nigral dopaminergic neurons, which are selectively activated in PD^15^,^16^,^17^ and membrane potential hyperpolarization might enhance iron transport function of DMT1, it is of vital importance to study the effects of activation of K_ATP_ channels on DMT1’s iron transport function. In the midbrain dopaminergic neurons, the K_ATP_ channels are composed of a pore-forming inward-rectifying potassium channel subunit, known as Kir6.2, and a regulatory sulfonylurea receptor subunit, known as SUR1^18^. These subunits are metabolic sensors that couple cellular energy metabolism to the membrane potential by regulating potassium efflux. SUR1 expression was selectively upregulated in nigral dopaminergic neurons in PD^15^,^17^. Some evidence has demonstrated that SUR1 mRNA expression was about two-fold higher in the nigral dopaminergic neurons than in ventral tegmental area (VTA) dopaminergic neurons in MPTP-induced PD models^15^. And the subunit SUR1 of K_ATP_ was selectively transcriptionally upregulated in human nigral dopaminergic neurons in PD patients. In contrast, mRNA expression of Kir6.2 was not altered^17^.

In the present study, to investigate the relationship between activation of K_ATP_ channels and DMT1-mediated iron transport function, using the quenching of calcein fluorescence indicated iron influx, we first observed the direct effect of activation of K_ATP_ channels on the iron transport function mediated by DMT1 in SK-N-SH cells. Then, in HEK293 cells, using patch clamp, we measured the changes of direct DMT1-mediated iron current by K_ATP_ channel activation. The effects of overexpression of K_ATP_ channels on iron influx were also investigated in SK-N-SH cells.

## Materials and Methods

### Chemical reagents

The SK-N-SH cells were from the Cell Bank of the Shanghai Institute of Cell Biology and Biochemistry, Chinese Academy of Sciences (Shanghai, China). The HEK293 cells and the JM109 bacterial strain were obtained from Dr. Yi-Ming Shao of Chinese Center for Disease Control and Prevention. The pcDNA3.1 vectors, which contained cDNA encoding SUR1 or Kir6.2, were a gift from Dr. Lily Yeh Jan, University of California, USA. Dulbecco’s modified Eagle’s medium (DMEM) was purchased from Gibco (Grand Island, NY, USA). Diazoxide, glibenclamide and FeSO_4_•7H_2_O were purchased from Sigma (St. Louis, MO, USA). Bisoxonol dye bis-(1, 3-dibutylbarbituric acid) trimethine oxonol (DiBAC_4_(3)), calcein-AM and carboxy-H_2_DCFDA were purchased from Molecular Probes (Eugene, OR, USA). Lipofectamine 2000 was purchased from Promega (Madison, Wisconsin, USA). All other chemicals and reagents were of the highest grade available and were purchased from local commercial sources.

### Cell culture and treatment

Human SK-N-SH neuroblastoma cells were cultured in Dulbecco’s modified Eagle’s medium (DMEM) supplemented with 10% fetal bovine serum (FBS), 100 U/mL of penicillin and 100 U/mL of streptomycin (pH 7.4) in a humidified atmosphere containing 5% CO_2_ at 37 °C. For experiments, cells were seeded in plates and grown to 80–90% confluency before they were treated with diazoxide (100 μM) or glibenclamide for 24 hrs. After 24 hrs, the medium was replaced with medium containing 100 μM Fe^2+^ and diazoxide or glibenclamide, and cells were treated for another 4 hrs and then harvested for experiments.

To observe the effect of activation or inhibition of the K_ATP_ channels on ferrous iron uptake and cell toxicity, the SK-N-SH cells were divided into three groups: a control group, a 100 μM Fe^2+^ group and a 100 μM Fe^2+^ and diazoxide or glibenclamide group. In the overexpression experiments, the SK-N-SH cells were seeded in plates and grown to a confluence of 70–80% and were then stably transfected with the pcDNA3.1 vector, which contained cDNA encoding SUR1 or Kir6.2 in serum-free medium. As a control, cells were transfected with the pcDNA3.1 vector alone. The cells were transiently cotransfected with vectors encoding SUR1 and Kir6.2 subunits at a molar plasmid ratio of 1:2. In the interference experiment, the SK-N-SH cells were transiently transfected with the pSilencer 4.1-DMT1. As a control, cells were transfected with the pSilencer 4.1 vector alone. All vectors were transfected using LipofectamineTM 2000. The ratio of lipofectamine to DNA for optimal transfection efficiency was 1:5. Additionally, the transfected cells were treated with 100 μM Fe^2+^ or 100 μM Fe^2+^ and diazoxide to observe the ferrous iron influx.

HEK293 cells (E1-transformed human embryonic kidney cells) were maintained in DMEM supplemented with 10% FBS, 100 U/ml of penicillin and 100 mg/ml of streptomycin at 37 °C in a humid (5% CO_2_, 95% air) environment. The HEK293 cells were resuspended in DMEM/F12 supplemented with 5% FBS and seeded at a density of 1 × 10^5^/ml 24 hrs prior to infection. When the confluence was approximately 50–70%, cells were infected with either AdGFP or AdDMT1 + IRE. GFP fluorescence was monitored at the indicated times to verify the infection efficiency. Thirty-six hours after infection, the cells were collected for use in further experiments.

### Membrane potential assay

The functional activity of the K_ATP_ channels was measured by evaluating the changes in membrane potential using the DiBAC_4_(3), a bis-barbituric acid oxonol with an excitation maximum of approximately 490 nm. Hyperpolarization results in extrusion of the dye and a decrease in fluorescence. Prior to the fluorescence measurements, the cells were incubated in HBS containing 5 μM of DiBAC_4_(3) for 30 min at 37 °C. The stained cells were used for experiments without washing. DiBAC_4_(3) fluorescence was recorded at an excitation wavelength of 488 nm and an emission wavelength of 525 nm, and the fluorescence intensity was measured every 1 min for 30 min. The mean fluorescence signal of 25–30 single cells in four separate fields was monitored at 200× magnification and processed with Fluoview 5.0 Software.

### Iron content assay

Inductively coupled plasma mass spectrometer (ICP-2) was used for the determination of the iron content. After treated with 100 μM Fe^2+^ or 100 μM Fe^2+^ and diazoxide, cells samples were washed with PBS for 3 times, digested with 1.5 ml nitric acid, then heat to obtain a limpid solution, constant volume to 2 ml at last. After 20 min of cooling at room temperature, samples were ready for iron content measurements.

### Calcein loading of cells and free iron levels assay

Calcein-AM is a membrane-permeative, non-fluorescent molecule that becomes fluorescent upon intracellular cleavage by cytoplasmic esterases, thereby forming calcein (which is membrane impermeative). This reaction is pH independent and stable and can be quenched rapidly by divalent metals and reversed easily by chelators. The ferrous iron influx into the SK-N-SH cells was determined by the quenching of calcein fluorescence^19^. The cells were incubated with calcein-AM (at a final concentration of 0.5 mM) in HEPES buffered saline (HBS, 10 mM of HEPES, 150 mM of NaCl, pH 7.4) for 30 min at 37 °C. The excess calcein on the cell surface was washed removed with three washes with HBS. The coverslips were mounted in a perfused heated chamber. Calcein fluorescence was recorded at an excitation wavelength of 488 nm and an emission wavelength of 525 nm and the fluorescence intensity was measured every 3 min for 30 min with continuous perfusion of 100 μM of ferrous iron (ferrous sulfate in an ascorbic acid solution, 1:44 molar ratio; prepared immediately before the experiments). Ascorbic acid maintained the reduced status of ferrous iron, in addition, ascorbate acted as a chelator to maintain the iron in solution. The mean fluorescence signal of 25–30 single cells in four separate fields was monitored at 200× magnification and processed with Fluoview 5.0 software.

### Whole-cell patch clamp recording

The HEK293 cells were planted on glass cover slips and cultured for 24 hrs until they reached 70–80% confluency and then co-transfected with DMT1 (with IRE) and K_ATP_ channel (SUR1 and Kir6.2 subunits). The positively transfected cells were identified by GFP fluorescence. The pipette and bath solutions used to measure DMT1–mediated cation currents were as described previously^20^. The bath solution contained 140 mM of NaCl, 10 mM of CaCl_2_, 10 mM of HEPES, 10 mM of MES, and 10 mM of glucose (pH 7.4). The pipette solution contained 147 mM of cesium, 120 mM of methanesulfonate, 8 mM of NaCl, 10 mM of EGTA, 2 mM of Mg-ATP, and 20 mM of HEPES (pH 7.4). The currents were recorded with an EPC-9 patch clamp amplifier (Heka Electronics, Germany) in the standard whole-cell configuration at room temperature. The currents were low-pass filtered at 2 kHz and sampled at 50 kHz. The patch pipettes were pulled from borosilicate glass capillaries (Sutter Instrument, Novato, CA) with a horizontal puller (P-97, Sutter Instrument). The pipettes typically had a resistance of 3–7 MΩ after being filled with an internal solution. After establishing the whole-cell mode, the capacitive transients and leakage currents were eliminated automatically. The whole-cell currents were elicited as described previously by repeated voltage ramps (−120 mV to +100 mV, duration 500 ms) from a holding potential of 0 mV^20^.

### Measurement of the mitochondrial transmembrane potential (ΔΨm) and the production of intracellular reactive oxygen species (ROS)

The changes in the mitochondrial membrane potential and the generation of ROS after various treatments in the SK-N-SH cells were measured with rhodamine123 or carboxy-H_2_DCFDA through flow cytometry (Becton Dickinson, USA) as previously described^21^.

### Cell viability assay

The cell viability were detected by the conventional 3-(4,5-dimethylthiazol-2-yl) -2,5-diphenyltetrazolium bromide (MTT) assay. The MTT assay relies primarily on mitochondrial metabolic capacity of viable cells and reflects the intracellular redox state. After incubation, then cells were incubated in MTT (5 mg/ml) for 4 hrs. The medium was removed and 100 μL DMSO was added to each well. The formazan dye crystals were solubilized for 10 min, and absorbance was measured by colorimetric assay (Molecular Device, M5, USA).

### Western blot analysis

The SK-N-SH cells were washed with ice-cold PBS and lysed in lysis buffer containing 50 mM of Tris HCl, 150 mM of NaCl, 1% Nonidet P-40, 0.5% sodium deoxycholate, 1 mM of EDTA, 1 mM of phenylmethylsulfonyl fluoride (PMSF), and protease inhibitors (1 g/mL of pepstatin, 1 g/mL of aprotinin, and 1 g/mL of leupeptin). The lysates were centrifuged at 12,000 × g for 10 min, and the protein concentration of the supernatants was determined with a Bradford assay kit (Bio-Rad Laboratories, Hercules, CA, USA). A total of 60 μg of protein was run on 12% SDS polyacrylamide gels and transferred onto PVDF membranes (100 mA, 30 min). After overnight blocking with 10% non-fat milk at 4 °C, the membranes were incubated with rabbit anti-SOD (1:200, BIOS, China). Blots were also probed with anti-β-actin monoclonal antibody (1:8,000, BIOS, China) as a loading control. The cross-reactivity was visualized using ECL western blotting detection reagents and analysed through scanning densitometry with a UVP image system.

### Statistical analysis

Results are presented as means ± S.E.M. One-way analysis of variance (ANOVA) followed by the Student-Newman-Keuls test was used to compare difference between means in more than two groups. The iron uptake experiment was carried out using two-way ANOVA followed by the Student-Newman-Keuls test. Data was presented as means ± S.E.M. A probability of *P* < 0.05 was taken to indicate statistical significance.

## Results

### Activation of K_ATP_ channels resulted in an increased free iron levels in the SK-N-SH cells

**Figure 1:**
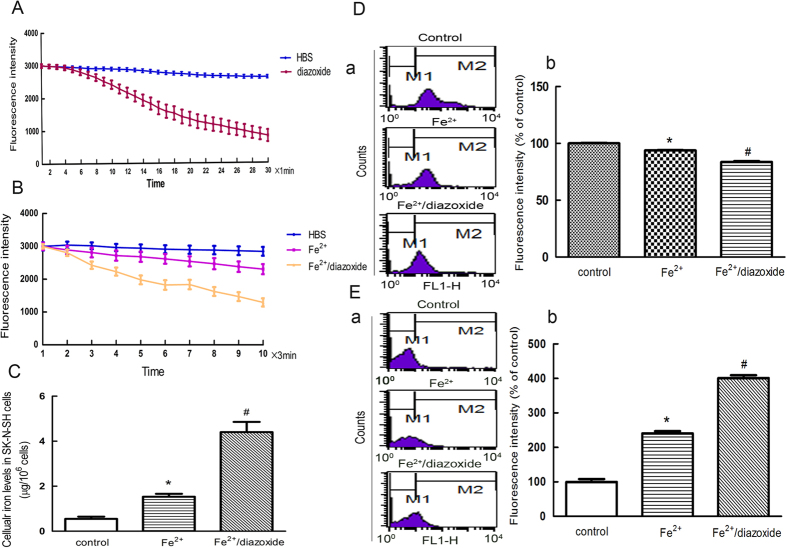
Activation of K_ATP_ channels resulted in hyperpolarization of the cell membrane and increased free iron levels in the SK-N-SH cells. (**A**) Diazoxide resulted in a graduated hyperpolarization of the membrane potential. Two-way ANOVA, *P* < 0.01, diazoxide compared with control, n = 8. (**B**) The cells treated with diazoxide showed more rapid and steady fluorescence quenching compared with that in the Fe^2+^ group. The fluorescence intensity is represented as the mean value of 35 separate cells from four separate fields at each time point and is presented as the mean ± S.E.M. of 6 independent experiments. Two-way ANOVA, *P* < 0.05, Fe^2+^ compared with control; *P* < 0.01, Fe^2+^/diazoxide group compared with Fe^2+^ group, n = 8. (**C**) Diazoxide treatment increased the intracellular iron concentration in the 100 μM diazoxide pre-treatment group compared with the Fe^2+^ group. *P < 0.05, compared with control; ^#^*P* < 0.01, compared with Fe^2+^ group, n = 6. (**D**) The increased cellular iron levels associated with a decrease in ΔΨ m. (a) Representatives of the fluorometric assay on ΔΨ m of different groups. Results are shown as FL1-H, setting the gated region M1 and M2 as a marker to observe the changing levels of fluorescence intensity using CellQuest software. The ΔΨ m was decreased in Fe^2+^-treated cells and the ratio of M2 area was decreased. Diazoxide could further decrease ΔΨ m. (b) Statistical analysis. Data was presented as mean ± S.E.M. of 6 independent experiments. Fluorescence values of the control were set to 100%. *P < 0.01 compared with control; ^#^*P* < 0.01, compared with the Fe^2+^ group, n = 6. (**E**) The increased cellular iron levels associated with increased ROS generation. (a) Representatives of the fluorometric assay on ROS generation of different groups. The ROS increased in Fe^2+^-treated cells and the ratio of M2 area was increased. Diazoxide could further increase the ROS generation. (b) Statistical analysis. Fluorescence values of the control were set to 100%. Data was presented as the mean ± S.E.M. of 6 independent experiments. *P < 0.01 compared with control; ^#^*P* < 0.01, compared with the Fe^2+^ group, n = 6.

To investigate whether activation of the K_ATP_ channels affects cellular iron transport, we measured the free iron levels in SK-N-SH cells treated with the novel K_ATP_ channel opener diazoxide. Treatment with 100 μM of diazoxide resulted in a gradual hyperpolarization of the membrane potential (Fig. 1A). The free iron levels in the SK-N-SH cells were subsequently measured by the quenching of calcein fluorescence, which is an indicator of intracellular iron levels. The cells treated with diazoxide showed a more rapid and steady fluorescence quenching compared with the Fe^2+^ group, indicating an increase in iron influx (Fig. 1B).

Next we measured the intracellular iron concentration using an inductively coupled plasma (ICP-2) detector. The results showed the intracellular iron concentration was increased in the 100 μM diazoxide pretreatment group compared with that of the 100 μM Fe^2+^ group (Fig. 1C). The increased intracellular iron levels resulted in cell damage, as indicated by changes in cellular ROS generation and ΔΨm. Compared with the 100 μM Fe^2+^ treated group, there was a 60% increase in the levels of ROS and a 154% decrease in the ΔΨm in the 100 μM Fe^2+^ and diazoxide group (Fig. 1D,E).

### DMT1 knockdown partially blocked influx of ferrous iron induced by the activation of K_ATP_ channels

**Figure 2:**
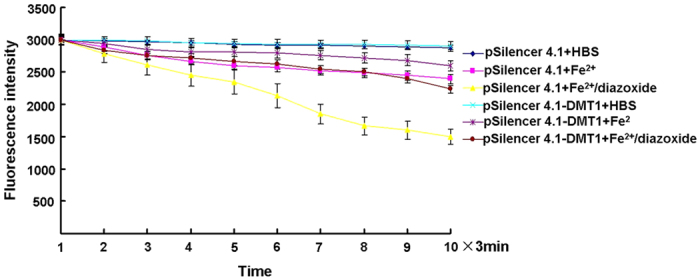
Influx of ferrous iron induced by activation of K_ATP_ channels was partially blocked by DMT1 knockdown. The fluorescence intensity is represented as the mean value of 35 separate cells from four separate fields at each time point and is presented as the mean ± S.E.M. of 6 independent experiments. Two-way ANOVA, *P* < 0.05 for the pSilencer4.1 group treated with Fe^2+^ compared with the pSilencer4.1 control group, and the pSilencer4.1-DMT1 group treated with Fe^2+^/diazoxide compared with the pSilencer4.1-DMT1 control group; *P* < 0.01 for the pSilencer4.1 group treated with Fe^2+^/diazoxide compared with the pSilencer4.1 group treated with Fe^2+^, and the pSilencer4.1-DMT1 group treated with Fe^2+^/diazoxide compared with the pSilencer4.1 group treated with Fe^2+^/diazoxide, n = 8.

To further determine whether the increased ferrous iron influx induced by the activation of the K_ATP_ channels was mediated by DMT1, pSilencer-DMT1 siRNA was used to knock down DMT1 levels in SK-N-SH cells. DMT1 expression levels were decrease by 55%, which could partially block iron influx by activation of the K_ATP_ channels (Fig. 2).

### Activation of K_ATP_ channels prolonged the iron-transport function of DMT1

**Figure 3:**
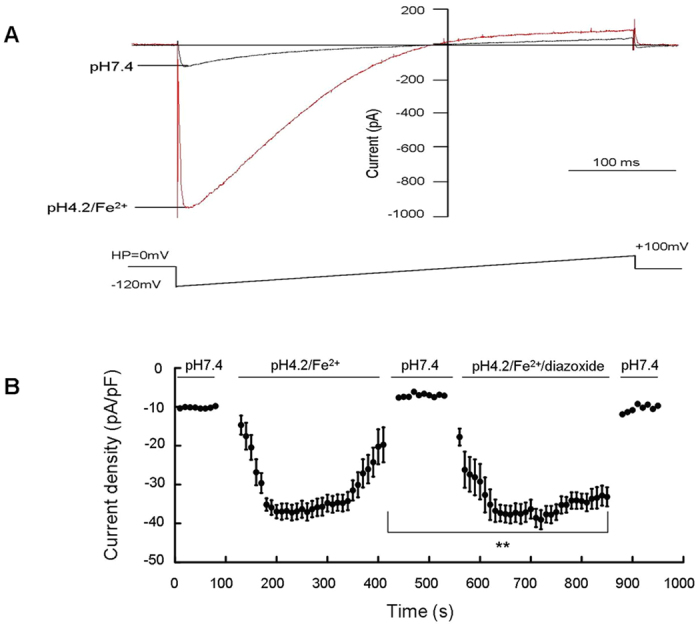
Activation of K_ATP_ channels by diazoxide delayed the current inactivation and prolonged the iron-transport function of DMT1. (**A**) DMT1-mediated Fe^2+^ current was evoked from voltage ramps. Whole-cell currents were elicited by repeated voltage ramps (−120 mV to +100 mV, duration 500 ms) from a holding potential of 0 mV. The time interval between ramps was 10 s. (**B**) Proton-dependent Fe^2+^ currents were measured in the absence and presence of 100 μM diazoxide. The solution exchange protocol is indicated above the current readings. **P < 0.01, between currents in the presence and absence of diazoxide with 100 μM Fe^2+^ at a pH 4.2, n = 8.

To give the direct evidence that this iron efflux was mediated by DMT1, next we measured the Fe^2+^-evoked currents by using whole cell patch clamp recordings. HEK293 cells were transfected with pcDNA3.1-SUR1 and pcDNA3.1-Kir6.2 vectors at a molar plasmid ratio of 1:2, and then infected with AdDMT1 + IRE. Consistently with results from a previous report^20^, the highest ferrous iron influx was observed between pH 5.5 to 6.5 in the experiments, whereas iron influx decreased to near baseline levels at pH 7.5 or above. We observed a rapidly activating, inwardly rectifying current in the DMT1-transfected HEK293 cells when the extracellular solution was changed from a control bath solution of pH 7.4 to an isotonic bath solution of pH 4.2, containing 100 μM Fe^2+^. After reaching a current density peak of −37.19 ± 4.61 pA/pF within 30 s after the solution exchange, this current was rapidly weakened in the continued presence of an acidic pH and Fe^2+^. In the presence of diazoxide (100 μM), a rapidly activating, inwardly rectifying current was also observed with a peak current density of −40.57 ± 5 pA/pF by Fe^2+^ at pH 4.2; diazoxide treatment reduced the current attenuation (Fig. 3). This result indicated the activation of the K_ATP_ channels by diazoxide delayed the current inactivation, and prolonged the iron transport activity of DMT1.

### Inhibition of the K_ATP_ channels blocked ferrous iron influx and the subsequent cell damage induced by Fe^2+^

**Figure 4:**
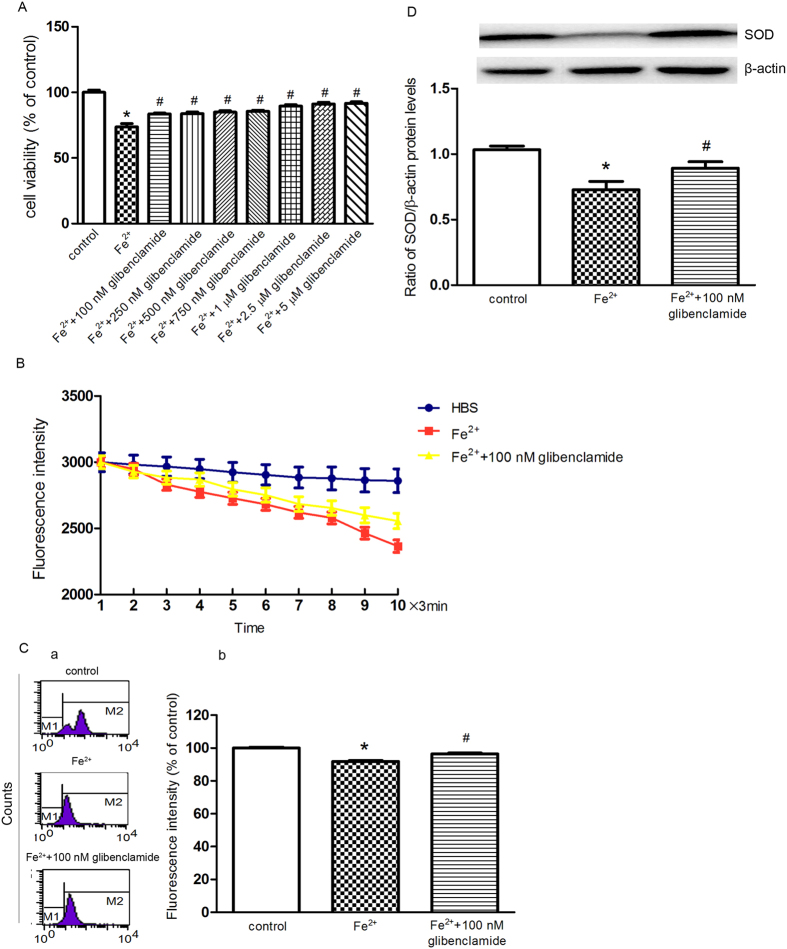
Inhibition of the K_ATP_ channels blocked ferrous iron influx and subsequent cell damage induced by Fe^2+^. (**A**) The changes in cell viability was observed with glibenclamide pre-treatment. The cell viability with seven doses of glibenclamide pre-treatment prior to Fe^2+^ treatment was determined by MTT assay. The data was presented as the mean ± S.E.M. of six independent experiments. *P < 0.01, compared with control; ^#^*P* < 0.01, compared with the Fe^2+^ group, n = 6. (**B**) The cells treated with 100 nM glibenclamide showed a more slow and steady fluorescence quenching, as compared with the Fe^2+^ group. The fluorescence intensities are represented as the mean value of 10 separate cells from four separate fields at each time point and are presented as the mean ± S.E.M. of 6 independent experiments. Two-way ANOVA, *P* < 0.05 for the Fe^2+^ group compared with control and the Fe^2+^/glibenclamide group compared with the Fe^2+^ group, n = 6. (**C**) Pre-treatment with 100 nM glibenclamide for 24 h partially reversed the decrease in ΔΨ m compared with the Fe^2+^ group. (a) Representatives of the fluorometric assay on ΔΨ m of different groups. (b) Statistical analysis. Data was presented as mean ± S.E.M. of 6 independent experiments. Fluorescence values of the control were set to 100%. *P < 0.01 compared with the control; ^#^*P* < 0.01 compared with the Fe^2+^ group, n = 6 (**D**) Pre-treatment with 100 nM glibenclamide for 24 h partially prevented the down regulation of Cu/Zn–SOD, as compared with the Fe^2+^ group. β-actin was used as a loading control. Data was presented as the ratio of Cu/Zn–SOD to β-actin. Data was presented as the mean ± S.E.M of six independent experiments. *P < 0.01, compared with control; ^#^*P* < 0.01, compared with the Fe^2+^ group, n = 6.

Alternatively, we investigated inhibition of the K_ATP_ channels on ferrous iron influx. The viability of cells showed a weak increase when cells were treated with 100 μM Fe^2+^ and glibenclamide (100 nM, 250 nM, 500 nM, 750 nM, 1 μM, 2.5 μM and 5 μM), respectively, compared with that of the 100 μM Fe^2+^ group, but there was no statistical significance between different doses of glibenclamide pretreatment (Fig. 4A). It has also been demonstrated that 100 nM glibenclamide could completely block the K_ATP_ channels directly^22^,^23^. Therefore, 100 nM glibenclamide was chosen for the following experiments. When the cells were perfused with 100 μM Fe^2+^ and 100 nM glibenclamide, the intracellular free iron levels were attenuated compared with that of the Fe^2+^ group (Fig. 4B). As presented in Fig. 4C, pre-treatment with 100 nM glibenclamide for 24 hrs partially reversed the decrease in ΔΨ m. Because SOD is a highly potent protective agent against cell injury during oxidative stress, we examined the levels of SOD in the SK-N-SH cells. In cells treated with 100 μM Fe^2+^, the Cu/Zn–SOD protein level was decreased by 23%, and this effect was reversed by pre-treatment with 100 nM glibenclamide (Fig. 4D).

### Overexpression of Kir6.2/SUR1 resulted in an increased free iron levels in SK-N-SH cells

**Figure 5:**
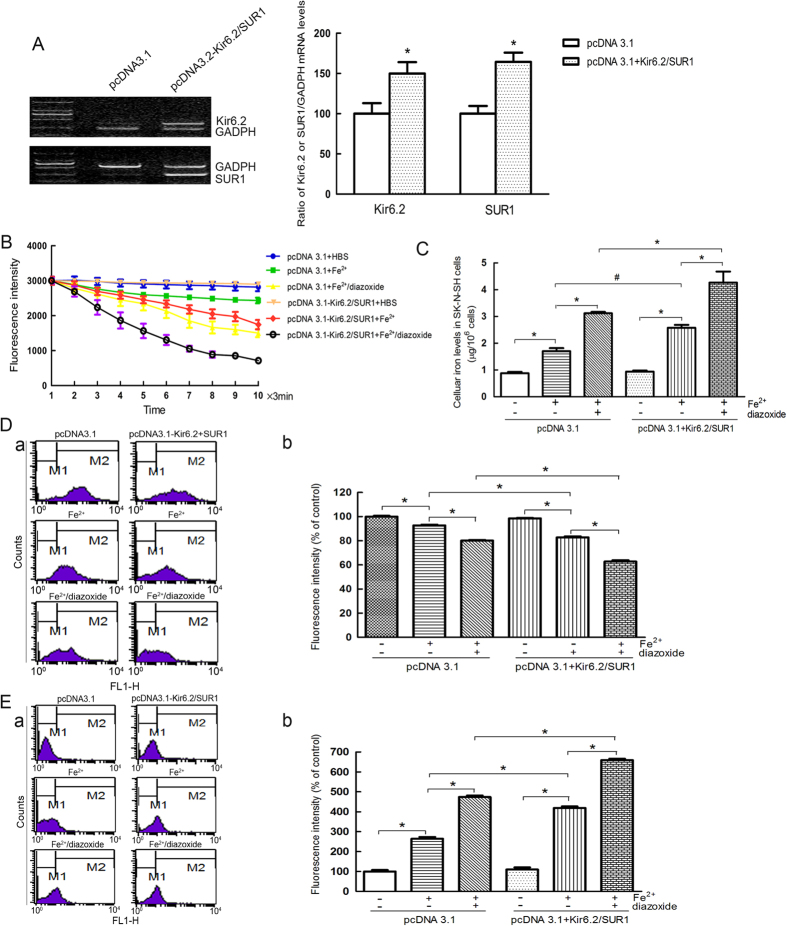
Overexpression of Kir6.2/SUR1 resulted in an increased free iron levels and cell damage. (**A**) The mRNA levels of kir6.2 and SUR1 in the SK-N-SH cells transfected with the pcDNA3.1-Kir6.2 and pcDNA3.1-SUR1 vectors. Data was presented as the mean ± S.E.M of 5 independent experiments. *P < 0.05, compared with the pcDNA3.1 group; n = 6. (**B**) The overexpression of K_ATP_ channels dramatically increased free iron levels. Two-way ANOVA, *P* < 0.05 for the pcDNA3.1 group treated with Fe^2+^ compared with the pcDNA3.1 group, and the pcDNA3.1-Kir6.2/pcDNA-SUR1 group treated with Fe^2+^ compared with the pcDNA3.1 group treated with Fe^2+^; *P* < 0.01 for the pcDNA3.1-Kir6.2/pcDNA-SUR1 group treated with Fe^2+^ compared with the pcDNA3.1-Kir6.2/pcDNA-SUR1 group, the pcDNA3.1-Kir6.2/SUR1 group treated with Fe^2+^/diazoxide compared with the pcDNA3.1-Kir6.2/pcDNA-SUR1 treated with Fe^2+^group, the pcDNA3.1-Kir6.2/pcDNA-SUR1 group treated with Fe^2+^/diazoxide compared with the pcDNA3.1 group treated with Fe^2+^/diazoxide, and the pcDNA3.1 group treated with Fe^2+^/diazoxide compared with the pcDNA3.1 group treated with Fe^2+^, n = 10. (**C**) The overexpression of K_ATP_ channels dramatically increased the intracellular iron concentration. *P < 0.01; ^#^*P* < 0.05, n = 6. (**D**) Overexpression of K_ATP_ channels further enhanced the iron-induced decrease in ΔΨ m. (a) Representatives of the fluorometric assay on ΔΨ m of different groups. (b) Statistical analysis. Data was presented as mean ± S.E.M. of 6 independent experiments. Fluorescence values of the control were set to 100%. *P < 0.01, n = 6. (**E**) The overexpression of K_ATP_ channels further enhanced the iron-induced increase in ROS generation. (a) Representatives of the fluorometric assay on ROS generation of different groups. (b) Statistical analysis. Fluorescence values of the control were set to 100%. Data was presented as the mean ± S.E.M of 6 independent experiments. *P < 0.01, n = 6.

Finally, pcDNA3.1-SUR1 and pcDNA3.1-Kir6.2 vectors at a molar plasmid ratio of 1:2 were transfected in the SK-N-SH cells to investigate the effects of overexpression of the K_ATP_ channels on the intracellular iron levels. There was a 1.5-fold increase in the expression of Kir6.2 and a 1.6-fold increase in the expression of SUR1 in these cells (Fig. 5A). The cells showed an increase in free iron levels when perfused with 100 μM Fe^2+^ (Fig. 5B), indicating that the overexpression of the K_ATP_ channels increased the iron uptake. Accordingly, the intracellular iron levels were also increased, which induced a decrease in ΔΨ m and an increase in ROS production, respectively (Fig. 5D,E). Consistently with previous results, diazoxide enhanced the intracellular iron uptake. The fluorescence intensity of the pcDNA3.1-SUR1/pcDNA-Kir6.2 group was decreased compared with that of the pcDNA3.1 vector group when perfused with 100 μM Fe^2+^ and with that of the diazoxide group, and the fluorescence intensity of the pcDNA3.1-SUR1/pcDNA-Kir6.2 group was decreased compared with that of the pcDNA3.1 vector group. The overexpression of SUR1 and Kir6.2, compared with that of the pcDNA3.1 vector alone, dramatically increased the free iron levels and iron content in the SK-N-SH cells treated with 100 μM diazoxide and Fe^2+^, which induced a decrease ΔΨ m and an increase in ROS production, respectively.

## Discussion

**Figure 6:**
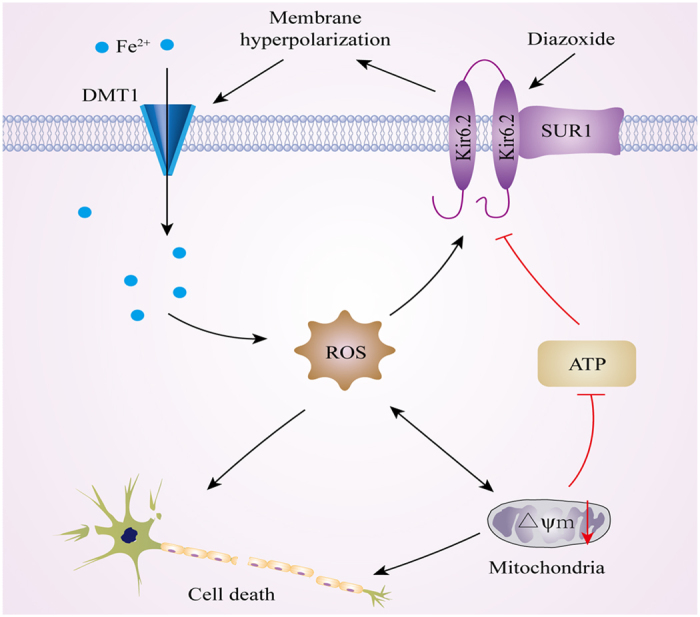
A schematic diagram of the functional relationship between the K_ATP_ channels and DMT1 in dopaminergic neurons. Activation of K_ATP_ channels caused hyperpolarization of the cell membrane, thus resulting in the enhanced iron transport function of DMT1. Iron accumulation in the cytosol caused an increase in oxidative stress, leading to the subsequent mitochondrial dysfunction and ATP depletion. This in turn led to activation of additional K_ATP_ channels in a feed-forward cycle.

Our findings showed that activation of the K_ATP_ channels in the SK-N-SH cells caused hyperpolarization of the cell membrane, which enhanced the iron uptake function of DMT1. This led to the increased intracellular iron levels and oxidative stress, and ultimately, cell death (Fig. 6). The inhibition of the K_ATP_ channels protected the SK-N-SH cells from the ferrous iron insult. This is the first study to link the function of the K_ATP_ channels and DMT1, which both contribute to the degeneration of nigral dopaminergic neurons in PD.

The precise mechanisms underlying the selective degeneration of nigral dopaminergic neurons of PD are unknown. Because of the role of iron in the generation of oxidative stress and protein aggregation, iron accumulation in the SN might be an important factor involved in the selective degeneration of dopaminergic neurons in PD. In addition, our previous studies have revealed that the selective iron accumulation mainly associate with increased expression of DMT1 in the SN in MPTP-induced PD models^13^. Furthermore, Salazar *et al.* also reported that higher DMT1 expression and consequently higher iron levels in nigral dopaminergic neurons might increase the vulnerability of these neurons to PD-related insults^12^. As reported, iron responsive element (IRE)- iron regulatory proteins (IRPs) systems, Ndfip1 and inflammation are all involved in the regulation of DMT1 expression^21^,^24^,^25^,^26^,^27^. Besides expression regulation, DMT1 iron transport function also dependents on H^+^ and membrane potential^12^,^14^,^28^. In *Xenopus* oocytes, Fe^2+^ transport rates are higher at hyperpolarized potentials and the maximal Fe^2+^-evoked current (I^Fe^) increases with hyperpolarization, showing a curvilinear dependence on Vm^14^. This indicated that DMT1-mediated iron transport might depend on the membrane potential, which increased with hyperpolarization.

Recently, K_ATP_ channels have been identified to render nigral dopaminergic neurons particularly vulnerable to degeneration in PD patients and in animal models^15^,^29^. K_ATP_ channels adapt the energy-demanding electrical activity of excitable cells according to their metabolic states, thus protecting neurons from overexcitability and excitotoxicity^30^. K_ATP_ channels in the nigral dopaminergic neurons are particularly sensitive to metabolic stress caused by PD-trigger factors, such as the mitochondrial complex I blockers, MPTP and rotenone. In Kir6.2 KO mice, a selective and complete rescue of adult nigral dopaminergic neurons had been observed in a chronic low-dose MPTP-induced PD model *in vivo*^31^. In addition, a further human study demonstrated the remaining nigral dopaminergic neurons from PD patients expressed two-fold higher levels of the K_ATP_ channel subunit SUR1, which was responsible for channel trafficking to the plasma membrane^17^. K_ATP_ channels triggered burst firing in mouse nigral dopaminergic neurons which may represent a compensatory response to the progressive loss of these neurons^17^,^32^. However, if the activities of the K_ATP_ channels were further increased in the nigral dopaminergic neurons (e.g., due to enhanced oxidative stress), the resulting hyperpolarization of the cell membrane would inevitably lead to reduced activity, or even silencing of the cells^15^. A reduction in nigral dopaminergic activity would be beneficial under metabolic stress in the short term. However, in the long term, the reduced electrical activity could be detrimental to these neurons.

In the present study, we showed activation of K_ATP_ channels in the SK-N-SH resulted in enhanced DMT1-mediated iron uptake in SK-N-SH cells, ultimately leading to cell death. Free iron is highly toxic because of its ability to generate free radicals; in particular, Fe^2+^ generates the highly toxic hydroxyl and superoxide free radicals in the presence of either hydrogen peroxide or molecular oxygen. Oxidative stress plays an important role in PD. Dopamine-rich areas of the brain are particularly vulnerable to oxidative stress because the metabolism of dopamine itself leads to the generation of ROS. In addition, the deficits of the mitochondrial respiratory chain and the ubiquitin–proteasome system in PD also lead to increase free radical formation. The inactivation of K_ATP_ channels blocks DMT1-mediated ferrous iron uptake, and the subsequent cell damage. Glibenclamide is a well-established and widely used drug to treat type 2 diabetes (T2D)^33^, and a retrospective meta-analysis has revealed epidemiological evidence of a reduced risk for PD in T2D patients treated with glibenclamide^34^,^35^,^36^.

## Conclusions

This work demonstrates that the activation of K_ATP_ channels enhances DMT1-mediated iron uptake in SK-N-SH cells, which may result from hyperpolarization of the cell membrane. The subsequent ATP depletion and ROS production induces the activation of additional K_ATP_ channels in a feed-forward cycle. The inhibition of the K_ATP_ channels dramatically decreases the iron uptake and inhibits cell damage. This study may provide new theoretical and experimental evidence for the role of K_ATP_ channels in iron accumulation and the degeneration of dopaminergic neurons in PD.

## Additional Information

**How to cite this article**: Du, X. *et al.* Activation of ATP-sensitive potassium channels enhances DMT1-mediated iron uptake in SK-N-SH cells *in vitro.* *Sci. Rep.* **6**, 33674; doi: 10.1038/srep33674 (2016).
